# Increasing the Density of Laboratory Measures for Machine Learning Applications

**DOI:** 10.3390/jcm10010103

**Published:** 2020-12-30

**Authors:** Vida Abedi, Jiang Li, Manu K. Shivakumar, Venkatesh Avula, Durgesh P. Chaudhary, Matthew J. Shellenberger, Harshit S. Khara, Yanfei Zhang, Ming Ta Michael Lee, Donna M. Wolk, Mohammed Yeasin, Raquel Hontecillas, Josep Bassaganya-Riera, Ramin Zand

**Affiliations:** 1 Department of Molecular and Functional Genomics, Geisinger Health System, Danville, PA 17822, USA; jli@geisinger.edu (J.L.); vavula1@geisinger.edu (V.A.); 2 NIMML Institute, Blacksburg, VA 24060, USA; rmagarzo@biotherapeuticsinc.com (R.H.); jbassaganya@biotherapeuticsinc.com (J.B.-R.); 3 Geisinger Medical Center, Biomedical Translational Informatics Institute, Danville, PA 17822, USA; manu.ksmanu@gmail.com; 4 Geisinger Medical Center, Neuroscience Institute, Danville, PA 17822, USA; dpchaudhary@geisinger.edu (D.P.C.); rzand@geisinger.edu (R.Z.); 5 Geisinger Medical Center, Department of Gastroenterology and Hepatology, Danville, PA 17822, USA; mjshellenberger@geisinger.edu (M.J.S.); hskhara@geisinger.edu (H.S.K.); 6 Geisinger Medical Center, Genomic Medicine Institute, Danville, PA 17822, USA; yzhang1@geisinger.edu (Y.Z.); mlee2@geisinger.edu (M.T.M.L.); 7 Molecular and Microbial Diagnostics and Development, Geisinger Medical Center, Danville, PA 17822, USA; dmwolk@geisinger.edu; 8 Department of Electrical and Computer Engineering, Memphis University, Memphis, TN 38152, USA; myeasin@memphis.edu; 9 BioTherapeutics, Inc., Blacksburg, VA 24060, USA

**Keywords:** imputation, electronic health records, machine learning, EHR, laboratory measures, medical informatics, inflammatory bowel disease, *C. difficile* infection, osteoarthritis, complex diseases

## Abstract

Background. The imputation of missingness is a key step in Electronic Health Records (EHR) mining, as it can significantly affect the conclusions derived from the downstream analysis in translational medicine. The missingness of laboratory values in EHR is not at random, yet imputation techniques tend to disregard this key distinction. Consequently, the development of an adaptive imputation strategy designed specifically for EHR is an important step in improving the data imbalance and enhancing the predictive power of modeling tools for healthcare applications. Method. We analyzed the laboratory measures derived from Geisinger’s EHR on patients in three distinct cohorts—patients tested for *Clostridioides difficile* (Cdiff) infection, patients with a diagnosis of inflammatory bowel disease (IBD), and patients with a diagnosis of hip or knee osteoarthritis (OA). We extracted Logical Observation Identifiers Names and Codes (LOINC) from which we excluded those with 75% or more missingness. The comorbidities, primary or secondary diagnosis, as well as active problem lists, were also extracted. The adaptive imputation strategy was designed based on a hybrid approach. The comorbidity patterns of patients were transformed into latent patterns and then clustered. Imputation was performed on a cluster of patients for each cohort independently to show the generalizability of the method. The results were compared with imputation applied to the complete dataset without incorporating the information from comorbidity patterns. Results. We analyzed a total of 67,445 patients (11,230 IBD patients, 10,000 OA patients, and 46,215 patients tested for *C. difficile* infection). We extracted 495 LOINC and 11,230 diagnosis codes for the IBD cohort, 8160 diagnosis codes for the Cdiff cohort, and 2042 diagnosis codes for the OA cohort based on the primary/secondary diagnosis and active problem list in the EHR. Overall, the most improvement from this strategy was observed when the laboratory measures had a higher level of missingness. The best root mean square error (RMSE) difference for each dataset was recorded as −35.5 for the Cdiff, −8.3 for the IBD, and −11.3 for the OA dataset. Conclusions. An adaptive imputation strategy designed specifically for EHR that uses complementary information from the clinical profile of the patient can be used to improve the imputation of missing laboratory values, especially when laboratory codes with high levels of missingness are included in the analysis.

## 1. Introduction

Given the complexity and high dimensionality of Electronic Health Records (EHR), the need for imputation is an inevitable aspect in any study that attempts to use such data for downstream analysis or building advanced machine learning models for decision support systems for clinical applications. The EHR or any other administrative dataset is not designed for research purposes, even though the breadth and depth of the information can be used to improve care at many levels [1]. Furthermore, the level and extent of the missing values in healthcare systems are typically not at random. Three main categories explain the missingness in clinical settings [2,3]—incompleteness, inconsistency, and inaccuracy—and these can capture a variety of situations, including the following: the patient could have been cared for outside of the healthcare system where the data are collected, the patient did not seek treatment, the health care provider did not enter the information, the patient expired, and the missing value was not needed. 

Given the complexity of the clinical data and the advanced analytics that can be applied on such data, it is important to account for any sources of bias in the data that will be used to drive predictive models. Imputation is an example of data preprocessing that could lead to biased results. Furthermore, excluding variables or patients with a high-level of missingness can also introduce bias and reduce the scope of the study. From a recent review article, 85 out of 316 studies reported some form of missing data, and only 12 studies actively handled the missingness; as the authors showed, the majority of researchers exclude incomplete cases, causing biased outcomes [4]. Furthermore, imputation could boost the statistical power for data-poor patients who tend to be minorities and low-income patients with more restricted access to primary and specialty care and rehabilitation programs.

Imputation has been an ongoing solution in many fields, but only recently, the research has been focused on medical applications. Twelve different imputation techniques applied to laboratory measures from EHR were compared [5]. In general, the authors found that Multivariate Imputation by Chained Equations (MICE) and softImpute consistently imputed missing values with low error [5]; however, in that study, the analysis was restricted to 28 most commonly available variables. In another study, the authors assessed the different causes of missing data in the EHR data and identified these causes to be the source of unintentional bias [6]. A comparative analysis of three methods of imputation (a Singular Value Decomposition (SVD)-based method (SVDimpute), weighted K-nearest neighbors (KNNimpute), and row average for DNA microarrays showed that, in general, KNN and SVD methods surpass the commonly accepted solutions of filling missing values with zeros or row averages [7]. However, comparing imputation for clinical data with a DNA microarray can be misleading. The missingness in a DNA microarray is likely at random due to technical challenges unlike missingness in the EHR. In another study, fuzzy clustering was integrated with a neural network to enhance the imputation process [8]. 

Research has also been done to evaluate imputation methods for non-normal data [9]. Using simulated data from a range of non-normal distributions and a level of missingness of 50% (missing completely at random or missing at random), it was found that the linearity between variables could be used to determine the need for transformation for non-normal variables. In the case of a linear relationship, transformation can introduce bias, while the nonlinear relationship between variables may require adequate transformation to accurately capture the nonlinearity. Furthermore, many of the techniques are optimized for smaller levels of missingness (the most commonly available measurements), yet most clinical datasets (including the EHRs) have a significant level of missingness for many of their important variables that are routinely used for diagnosis purposes. To address this problem, machine learning methods have also been proposed [10]. There are more examples of imputation applied to simulated than real-life EHR data; however, few studies focused on imputing laboratory values. For instance, Ford E. and colleagues [11] proposed using logistic regression models with and without Bayesian priors representing the rates of misclassification in the data. However, in that study, the authors focused on misclassified diagnoses rather than laboratory values. The challenges of imputation for EHRs are unique, and if left unaddressed, the utility of the data becomes limited [12]. Consequently, even though, for smaller targeted studies, it could be possible to integrate additional modalities or perform an analytical evaluation through a chart review to determine a likely cause of missingness, for larger studies, this becomes infeasible. For example, the missingness level for very important variables, such as hemoglobin A1C or HbA1c (LOINC ID: 17856-6) levels, a common biomarker for diabetes can easily reach 50% or more in many realistic large datasets. At last, in a more recent study, the integration of genetic and clinical information was shown to improve the imputation of data missing from the Electronic Health Records [13]; however, genetic data integrated with the EHR is still scarce.

Finally, given the complexity and the scale of the problem, in many studies, MICE [14] remains the method of choice. The MICE fully conditional specification (FCS) algorithm imputes multivariate missing data on a variable-by-variable basis [15]. An imputation model is specified for each incomplete variable, and the imputation of missingness in one variable is conducted iteratively based on the other variables. There are also variations of MICE that have been proposed [16]; however, the need for imputation for data from EHR poses its challenges, especially when targeting less commonly measured variables. Nonetheless, given the high level of redundancy and the presence of highly correlated entities in the EHR, imputation by MICE still performs relatively well for large clinical datasets. A comprehensive overview of handling missing data in the EHR is presented in [12].

In this study, we created three unique cohorts from the EHR data, with varying sizes and heterogeneity, and developed a hybrid imputation strategy that we applied to these cohorts. We selected the inflammatory bowel disease cohort because of its heterogeneity and the fact that a clear understanding of IBD’s risk factors is still lacking. We selected the *Clostridioides difficile*, because understanding of the recurrent infection is important, and the existing data from the EHR can help us identify clinical biomarkers; finally, we created the osteoarthritis (OA) cohort to test the limits of this model, as the OA diagnosis is not based on any laboratory measurements known today. Our imputation model was based on using comorbidity information to cluster patients prior to the imputation of their laboratory values.

## 2. Methods

In the following section, we will (1) describe our cohort definition and data extraction for the laboratory values and comorbidities from our EHR data warehouse and (2) outline our imputation design.

### 2.1. Study Cohort

The cohort in this study consisted of 67,445 patients from the Geisinger Health System with three different phenotypes. This study was exempted by the Geisinger Institutional Review Board for using deidentified information.

*Clostridioides difficile* (Cdiff) Infection case and control cohort: *Clostridioides difficile* (*C. difficile*) is an anaerobic, Gram-positive, and spore-forming bacterium and a major cause of intestinal infection and antibiotic-associated diarrhea. Toxins are the major virulence factors of *C. difficile* [17]. Toxins A (TcdA) and B (TcdB) are large, secreted glucosyltransferase proteins that target intestinal epithelia cells and disrupt the epithelial barrier, leading to secretory diarrhea. The diagnosis of *C. difficile* at Geisinger is captured and documented by Polymerase Chain Reaction (PCR) confirmation, which is highly sensitive. The latter is also considered the gold standard by the eMERGE algorithm for EHR mining [18]. We identified the *C. difficile* cohort, which includes patients tested for *C. difficile*, from the EHR of the Geisinger Health System. The cohort includes both cases and controls. Cases are defined as having laboratory positive PCR test results. Controls are patients tested for *C. difficile* with negative PCR test results. Case/control ratio is 1:8. We are interested in the combined case and control cohort, since patients tested for *C. difficile*, irrespective of their test results, share some of the signs and symptoms (such as diarrhea); furthermore, using a case and control combined cohort increases our sample size, an important factor for imputation, while providing a framework for building predictive models that can benefit from the integration of a large number of laboratory-based features.

Inflammatory Bowel Disease (IBD) cohort: We identified the IBD cohort from the EHR of the Geisinger Health System. Inclusion criteria of this cohort were based on the extraction of the patient population based on the diagnosis recorded for patients under their visits, admissions, and currently active problems listed based on the ICD9 and ICD10 codes for Crohn’s disease (CD) and ulcerative colitis (UC) (see Table A1 in Appendix A). To have a higher fidelity regarding the diagnosis in the EHR, qualifying criteria included either two or more outpatient encounters, or one or more inpatient admissions, or an entry into the problem list with an active flag.

Osteoarthritis (OA) cohort: We identified an osteoarthritis (OA) cohort from the EHR of the Geisinger Health System; the cohort includes a knee or hip OA diagnosis, either primary or secondary diagnosis (see Table A1 in Appendix A for the OA diagnosis ICD codes).

### 2.2. Data Extraction

We extracted clinical laboratory measurements for this cohort using the Logical Observation Identifiers Names and Codes (LOINC) system. For comorbidities, we extracted all the diagnosis codes for all the patients based on the ICD9, as well as ICD10, codes. Comorbidity data included details from out-patient visits, in-patient admissions, and problem lists. The latter was used to capture conditions identified outside of the Geisinger Health System but discussed and assessed during the patient’s care management. We excluded laboratory codes with more than 75% missingness. To further clarify, in this study, missingness is defined as the laboratory measure “not resulted”. Therefore, if an order was placed but the results were not available (or not valid), we considered that as a missing value. We analyzed the data in three batches, including only laboratory measures that have, at most, (a) 25% missingness, (b) 50% missingness, and (c) 75% missingness.

### 2.3. Data Processing

Quality Control (QC) and outlier detection strategy: Geisinger has implemented a rigorous process to continuously extract, transform, organize, and store EHR data and remove erroneous entries for research purposes. For example, we currently have access to quality-controlled laboratory values with the reconciliation of units. Median laboratory values for each patient were calculated to be used for this study. It is important to mention that, especially for less common laboratory values, the frequency of measurements and the window between the first and last measurements per patient is relatively narrow. We analyzed the frequency patterns and reported the results in our descriptive section.

As part of the added data processing and outlier detection and removal, the distribution of each laboratory value was analyzed and fit to a tri-modal gaussian distribution model (see Equation (1)). The rationale for using this strategy, as opposed to the assumption of normality, is driven by the nature of the laboratory measures. Laboratory orders, especially those with a higher level of missingness, are typically missing not at random (MNAR), and there are mainly three groups of patients for whom there is a measurement recorded (those with higher or lower than average measures, as well as patients with average measurements). However, the average measurement is not necessarily associated with a larger group in all the cases, especially for laboratory measures that are specific to a phenotype, such as an iron-binding capacity. The latter is ordered for patients if the physician needs that information to make a diagnosis/management decision. Two cut-off values are created to filter outliers based on the three distributions model. The automated process to generate data-driven cut-off values is proposed for large-scale data mining, where limited manual curation is applied in the data preparation and preprocessing.

(1) $$f=N_{1}\left(\mu_{1},\sigma_{1}^{2}\right)+N_{2}\left(\mu_{2},\sigma_{2}^{2}\right)+N_{3}\left(\mu_{3},\sigma_{3}^{2}\right)$$

where *μ* is the mean and *σ* is the standard deviation. The lowest boundary to filter out the outliers is set to *c_low* = max (min(*μ*_1_ − 3*σ*_1_,*μ*_2_ − 3*σ*_2_, *μ*_3_ − 3*σ*_3_), 0), and the highest boundary is set to *c_high* = max(*μ*_1_ + 3*σ*_1_,*μ*_2_ + 3*σ*_2_, *μ*_3_ + 3*σ*_3_).

Data processing of the comorbidity dataset was performed to remove noise by excluding the ICD9/10 codes that were recorded only once in the patient’s chart (rule of 2). The resulting matrix was then converted to binary to represent the presence or absence of an ICD9/10 code for each patient. This is important, since the count does not necessarily correlate with the severity or duration of the condition. Therefore, a binary comorbidity matrix for each cohort was created for imputation modeling.

### 2.4. Data Abstraction and Imputation Strategy

The comorbidity dataset was used to compute an encoding matrix for each dataset (Cdiff, OA, and IBD) using singular value decomposition (Equation (2)).

(2) $$A_{PT\_ICD\_cohort}=A_{PT\;X\;ICD\_cohort}=USV^{T}$$

where *A_PT_ICD_cohort_* is the matrix encompassing all the ICD9/10 codes (presence of absence) for all the patients for each dataset, *U* is an *mxm* square matrix, *S* is an *mxn* diagonal matrix with *m* rows and *n* colums, and *V* is an *nxn* square matrix. The columns of *V* are eigenvectors of *A^T^A*, and the columns of *U* are eigenvectors of *AA^T^*. The diagonal elements of *S* are the square root of the eigenvalues of *A^T^A* or *AA^T^*.

The encoding matrix was then used to create different levels of data abstraction by retaining only 100 or 1000 of the encoding using the dimensionality reduction technique (Equation (3)) for each dataset. We used these predefined cut-off values based on our preliminary assessment [19], as well as empirical studies [20,21]. For comparison, the full rank was also used in the modeling. Note that the approximation matrix is referred to as the data abstraction. The finalized output is referred to as latent comorbidities.

(3) $$A_{PT\_ICD\_g}=U_{reduced}S_{reduced}V_{reduced}^{T}$$

where *g* is the level of abstraction (100 or 1000) corresponding to the level of reduced matrices. *A_PT_ICD_cohort_g_* is an approximation of the initial matrix (*A_PT_ICD_cohort_*).

As a final step in the data abstraction process, a baseline noise reduction is performed by removing the ICD codes if the sum of all the values for a given code in the latent comorbidity matrix is less than 1. This strategy reduces noise that is due to irrelevant (very rare) comorbidities in the model. The imputation method presented in this work is a hybrid method—that is, based upon concurrently applying dimensionality reduction and a clustering strategy—to efficiently capture relationships among the features (or variables) and reduce noise (through dimensionality reduction) while providing an adaptive mechanism to perform imputation for any complex phenotype or trait. Using latent comorbidity data, patients are clustered using the k-mean clustering technique with *K* set to 2, 4, 8, and 16 clusters, depending on the heterogeneity of the cohort.

Imputation was applied using the MICE fully conditional specification (FCS) algorithm [5], which imputes multivariate missing data on a variable-by-variable basis. An imputation model is specified to each incomplete variable, and the imputation of missingness in one variable is conducted in an iterative fashion using the Markov Chain Monte Carlo (MCMC) method. More specifically, we selected the predictive mean matching (pmm) algorithm, which is the default method of mice() for imputing continuous incomplete variables. For each missing value, pmm finds a set of observed values (default is 5) with the closest predicted mean as the missing one and imputes the missing value by a random draw from that set. In other words, pmm is restricted to the observed values. We also used Random Forest (rf), which is based on imputing missingness by recursively subdividing the data based on values of the predictor variables in the predictive model by a bootstrap aggregation of multiple regression trees to reduce the risk of overfitting and improve the predictions through a combination of prediction from many trees [22]. The latter does not rely on distributional assumptions and can better accommodate nonlinear relations and interactions.

Imputations using MICE-pmm and MICE-rf were applied to each subgroup independently to predict the missing values. The results were compared when MICE-pmm and MICE-rf were applied to estimate the missing in the laboratory values in three cohorts without any consideration of the comorbidity information. The reader is referred to the work [15] by S. van Buuren and K. Groothuis-Oudshoorn for more details about imputation by MICE.

### 2.5. Evaluation Strategy

Model evaluation is performed by randomly selecting variables and predicting them using the hybrid strategy. A total of 100 values from each laboratory measure was randomly withheld for testing. For example, for the Cdiff cohort, where we identified 48 laboratory codes with less than 75% missingness, we held out 100 values for each of the 48 laboratory codes and estimate these 10 times. The root mean square error (RMSE) was also calculated and averaged over the 10 runs. Comparison was based on calculating the difference between running imputation using the hybrid model and the standard MICE algorithm, without any consideration of the comorbidity information, using both the pmm and rf models implemented in the MICE package. The presented results were, therefore, the RMSE differences, where the negative values represent a reduction in the root mean square error.

## 3. Results

In the following section, we will (1) describe our cohorts, pattern of missingness, and frequency of available data for different levels of missingness and (2) present imputation results for the three datasets.

### 3.1. Description of Laboratory Values for the Three Cohorts

We identified a total of 67,445 patients in three different cohorts (Cdiff, OA, and IBD) from Geisinger’s electronic data warehouse. Further, we identified 495 LOINC codes from this cohort. We selected the LOINC codes for which we had, at most, 75% missingness (i.e., the number of patients without any measurement divided by the total number of patients is less than or equal to 75%) in each of the three cohorts.

We identified a total of 46,215 patients tested for *C. difficile*. We extracted comorbidity and laboratory data from the EHR for this cohort. A total of 48 laboratory codes and 8160 ICD codes for comorbidities were used. Specifically, we identified a total of 48 of the laboratory codes from the 495 codes that had at least 25% of the 46,215 patients with at least one measurement in their records. It is important to highlight that many of the LOINC codes can be very specific (<1% of the patients have such measurements) or were used for a narrow period and may not be actively in use. The dimensionality reduction was set to 100 and 1000. The Cdiff cohort had high heterogeneity, since the dataset contained both cases (tested positive for *C. difficile*) and controls (tested negative for *C. difficile*). The number of clusters tested was 4, 8, and 16.

Similarly, we further identified 11,230 IBD patients with both comorbidity and laboratory data from the EHR. A total of 48 laboratory codes and 7916 ICD codes for comorbidities were identified. The dimensionality reduction was set to 100 and 1000. The number of clusters tested was two, four, and eight, given the smaller sample size of this cohort.

Finally, we identified 187,040 patients with a primary or secondary diagnosis of the knee or hip OA from which we randomly selected 10,000 patients for imputation modeling. A total of 44 laboratory codes and 2042 ICD codes for comorbidities were used. The OA cohort had high heterogeneity, since the dataset was large (almost 200,000 cases from the initial pool) and contained both hip and knee OA. We selected a random set of 10,000 patients, as it is impractical to use an extremely large cohort of patients for optimizing an imputation, as the optimization alone is a computationally extensive process. The number of clusters tested was 4, 8, and 16.

**Figure 1:**
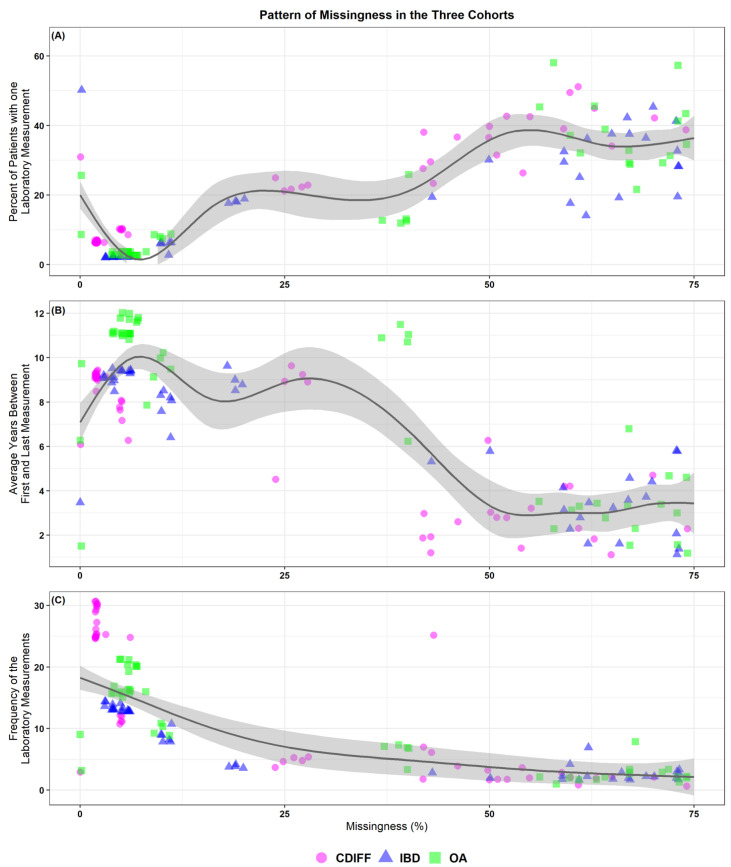
The pattern of missingness for the three cohorts. A generalized additive model was used for smoothing. The gray area around the smoothing curve represents a 95% confidence interval. (**A**) The percentage of patients with one laboratory measurement versus the missingness percentage for the three datasets. (**B**) The average number of years between the first and last laboratory measurements (calculated for patients with two or more measurements) versus the missingness percentage for the three datasets. (**C**) The frequency of the laboratory measurements calculated for patients with two or more measurements versus the missingness percentage for the three datasets. Cdiff: *Clostridioides difficile*, IBD: inflammatory bowel disease, and OA: osteoarthritis.

The distribution of missingness in the laboratory values was different for the different cohorts. Table A2 summarizes the percentage missing for the laboratory measures. Our results showed that the pattern and frequency of the laboratory measurements were dependent on the missingness level. Briefly, for laboratory values with high missingness, a larger percentage of patients (30–60%) had only one resulted value; therefore, the median that we calculated in our experiment was practically the exclusively reported value for the patient (see Figure 1A). We further observed that the laboratory values with a high level of missingness (when a patient had more than one value) tended to have an observation window of approximately two to six years (see Figure 1B) and a frequency that was below five measurements (see Figure 1C). However, for more common laboratory values, we observe a window of approximately 5 to 12 years and a frequency above 10 (see Figure 1C).

**Figure 2:**
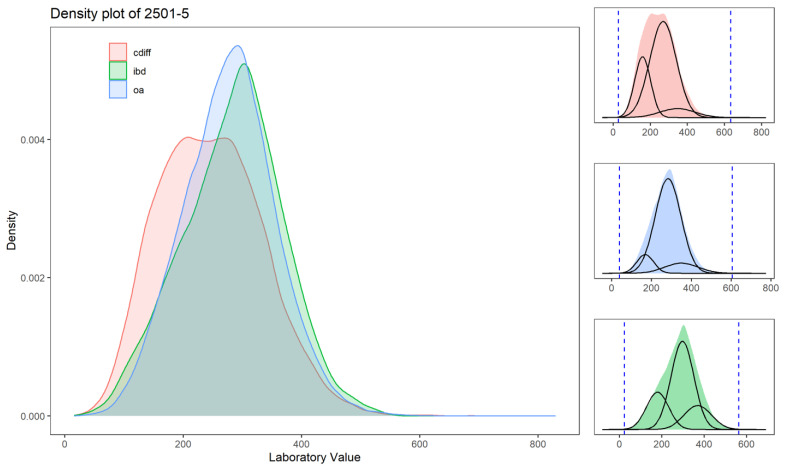
Distribution of laboratory values normalized for Logical Observation Identifiers Names and Codes (LOINC) 2501-5 (iron-binding capacity) for the three datasets (Cdiff in red, IBD in green, and OA in blue). The “ironbinding capacity” is missing at 52% in the Cdiff dataset, 65% in the IBD dataset, and 64% in the OA dataset. The subpanels represent the three modeled distributions to calculate the upper and lower boundaries. The dashed lines represent the upper and lower outlier boundaries (based on Equation (1)).

**Figure 3:**
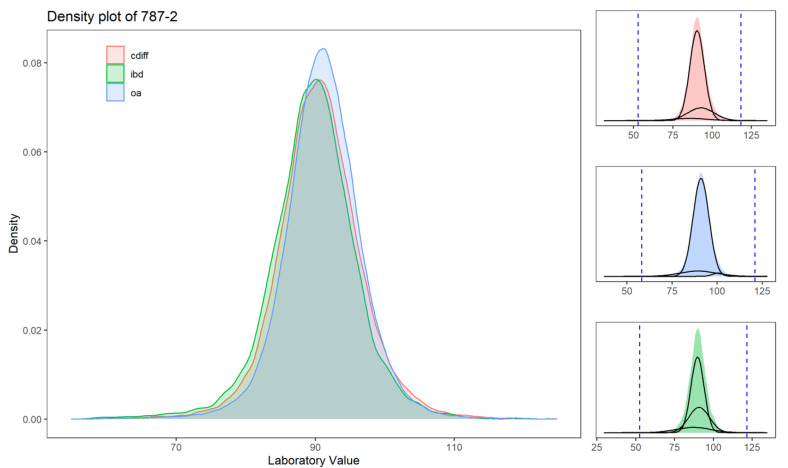
Distribution of laboratory values normalized for LOINC 787-2 (mean corpuscular volume or MCV) for the three datasets (Cdiff in red, IBD in green, and OA in blue). The “MCV” is missing at 2% in the Cdiff dataset, 5% in the IBD dataset, and 4% in the OA dataset. The subpanels represent the three modeled distributions to calculate the upper and lower boundaries. The dashed lines represent the upper and lower outlier boundaries (based on Equation (1)).

**Figure A1:**
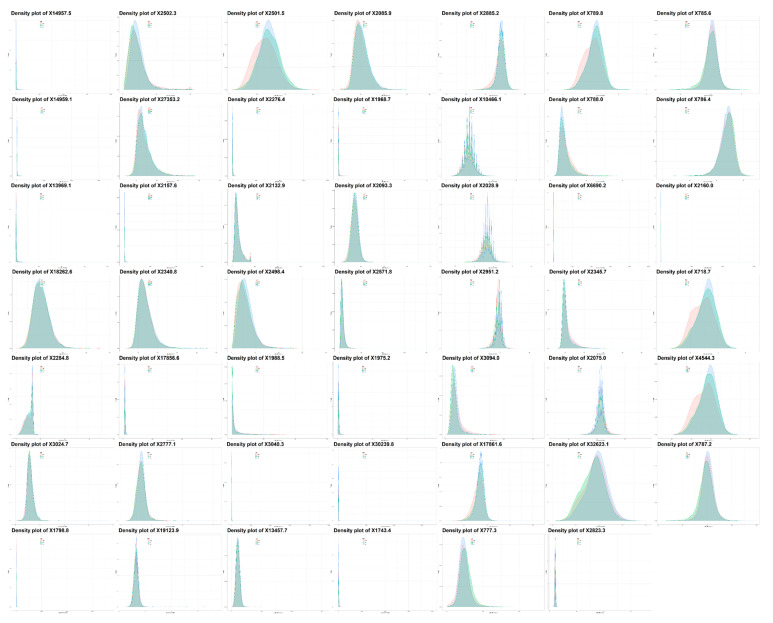
Distribution of the laboratory values normalized for all the LOINC included in this study.

The outlier detection using a multimodal gaussian distribution function was applied to each laboratory measure for each cohort separately. Figure 2 highlights that, for laboratories with higher missingness levels, the distribution is different for the different cohorts, and therefore, the accepted range is adjusted accordingly. For more common laboratory measures (such as the example presented in Figure 3), the distributions are similar. The accepted range for these laboratory measures is within the calculated range. To further help the reader to better understand the pattern of laboratory data, we created distribution plots for all the laboratory values used in this study for the three cohorts (see Figure A1 and Table A2).

### 3.2. Imputation Applied to Laboratory Values

**Figure 4:**
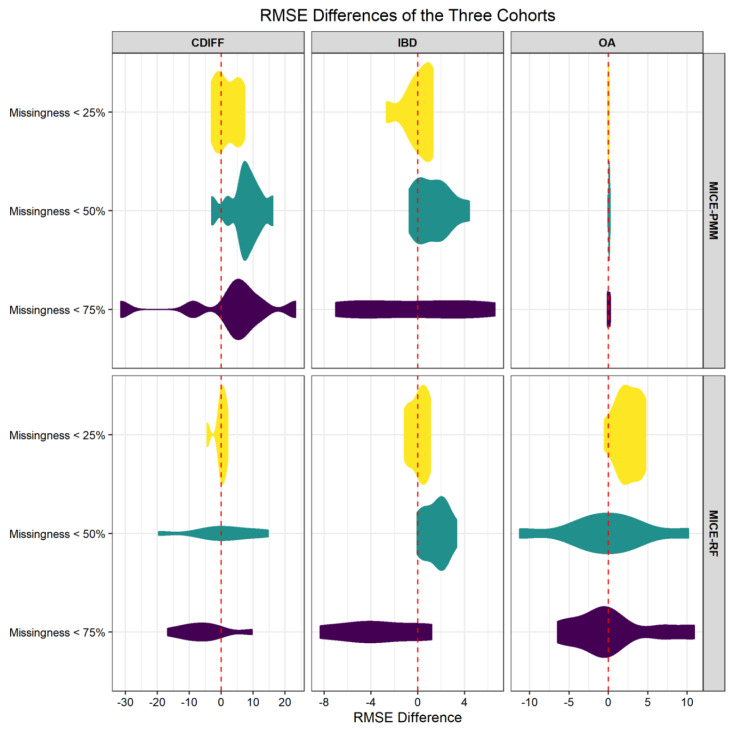
Violin plots representing the root mean square error (RMSE) differences—comparing the performance of Multivariate Imputation by Chained Equations (MICE) with and without the comorbidity information. Two algorithms, predictive mean matching (pmm) and Random Forest (rf), were compared. A Negative RMSE difference indicates a performance improvement when the comorbidity information is utilized.

*C. difficile* (Cdiff) infection case and control cohort: Using adaptive imputation for the Cdiff cohort showed improved performance, especially for the high missingness group (laboratory measures that have, at most, 75% missingness). An average RMSE difference (comparing the proposed imputation with the standard imputation model, without any consideration of comorbidity information using MICE) was −31.47 for a level of abstraction *g* = 1000 and a cluster number *k* = 4. The average RMSE difference was −8.75 for *g* = 100 and *k* = 4, demonstrating that, at a high missingness level, additional information from the patient comorbidity information can play an important role in improving the accuracy of the imputation prediction. A total of 27 combinations (or nine combinations for each missingness threshold) were tested, and for each missingness level (Table 1), the tradeoff between the sample size and clustering approach resulted in one or two instances where clustering was associated with improved performance. Since the dataset is of fixed size, the higher number of clusters will reduce the power of the imputation method, especially when the number of clusters is increased to eight or beyond. However, as each dataset has its unique characteristics, the best set of parameters must be empirically determined prior to performing the imputation using the adaptive strategy. Using MICE and the random forest model (rf), the RMSE differences were negative for the majority of the combinations. The missingness group of <75% had seven out of the nine parameter combinations that were in favor of the novel method (See Table 1 and Figure 4).

Inflammatory Bowel Disease (IBD) cohort: Using adaptive imputation for the IBD cohort showed improved performance, especially for the high missingness group (laboratory measures that have, at most, 75% missingness). An average RMSE difference when compared to the standard model using MICE alone was −8.35 with no abstraction and cluster number *k* = 2. Similarly, an average RMSE difference when compared to the standard model using MICE alone was −8.24 for *k* = 8. The results highlighted that, at a high missingness level, additional information from the patient comorbidity data can play an important role in improving the accuracy of the imputation prediction, even as the sample size is significantly smaller (in this case, 11 K versus 46 K for the Cdiff cohort). A total of 27 combinations (or nine combinations for each missingness threshold) were tested. The tradeoff between the sample size and clustering approach resulted in parameter combinations that were associated with improved performance. Additional analyses were performed with the random forest model in MICE, and an RMSE difference of −2.70 was recorded for a missingness level of 25% (see Table 1 and Figure 4). Our results corroborate the value of parameter optimization on the dataset using various modeling frameworks. Thus, the best set of parameters should be empirically determined for each dataset.

Osteoarthritis (OA) cohort: Using adaptive imputation for the OA cohort showed that the best performance improvement was for missingness at 50% (Table 1 and Figure 4). The tradeoff between the sample size reduction, when clustering is utilized, and the use of additional information from comorbidities did show benefits even for this smaller and more heterogeneous dataset. The rf model in MICE was best fitted for this dataset.

## 4. Discussion

This study is a first step towards improving our many layers of data analytics and quality control pipelines to help enhance the quality of data extracted from the EHR that is ingested in machine learning applications for precision medicine. The use of heterogeneous and large-scale clinical datasets, such as EHRs, provides an avenue for the exploration of strategies to improve care at individualized levels, which include developing personalized models of responses to therapy and the prediction of disease onset, among others [1]. However, the data extracted from EHRs are noisy and have many missing values. In the majority of studies, variables suffering from missingness are excluded from models and analyses [4], even for some variables with high discriminative ability according to the clinical knowledge. As we showed in this work, it is not recommended to solely rely on the redundancy of EHR laboratory data to conduct imputation for realistic applications. That is because the majority of redundancy from laboratory measurements are associated with variables that are missing at high levels. However, laboratory data is highly associated with comorbidity, as the latter is based on laboratory values in realistic settings. For instance, besides the commonly ordered laboratory tests (20–30 laboratory measures), the remaining values are missing at very high rates, even in a healthcare system with a stable population (Geisinger is an integrated healthcare system with a drop-out rate <5%). However, the laboratory measures are highly correlated with comorbidities and diagnosis. Therefore, our intuitive modeling strategy is focused on using this redundancy to improve the imputation for laboratory values.

Furthermore, many diagnoses are based on laboratory values; however, due to the challenges associated with mining laboratory measures, many models ignore this important parameter or only include the ones that are not missing at high levels to reduce the noise and bias due to poor imputation predictions. We created three diverse datasets to test this intuitive strategy of imputation designed specifically for EHR laboratory data by including information from the comorbidities.

The IBD dataset was used, because IBD is a heterogeneous disease and a clear understanding of its risk factors is still lacking. Recent advances in the knowledge of IBD’s pathogenesis have led to the implication of a complex interplay between metabolic reprogramming and immunity [23]. Furthermore, the response to treatment in IBD varies significantly among individuals and disease subtypes based on demographic characteristics, diet, comorbidities, underlying immunological factors, and genetic polymorphisms. Thus, there is an urgent unmet need to replace the current imputation approaches with personalized strategies that consider individual variability, diversity, and more balanced patient representation. Therefore, building predictive models for treatment outcomes for IBD is an important step in utilizing the available data on drug responses to provide better care for this patient population. Thus, the integration of laboratory measures in a predictive model for IBD has clinical value.

We created the Cdiff dataset, because the understanding of recurrent *C. difficile* infection is important, and the existing data from EHR can help us identify clinical biomarkers and help in building a decision support system for physicians to target the patients at a higher chance of recurrence for more targeted preventive care.

Finally, the OA dataset was added to test the limits of this model. An OA diagnosis is not based on any laboratory measure known today. An OA diagnosis is based on imaging alone. Therefore, we did not expect the OA cohort to have any special patterns in their laboratory profile, yet we observed that, even in this situation, the use of a comorbidity pattern can help in improving the imputation of laboratory values. The OA dataset was also the smallest dataset tested in this study.

Overall, our results showed that each dataset is unique, and a one-size-fits-all approach does not apply when selecting the imputation model. On simulated datasets with interactions between variables, the imputation of missing data using MICE with regression trees resulted in less biased parameter estimates than MICE with linear regression. [24] In the CALIBER study, MICE random forest showed more imputation efficiency with narrower confidence intervals for the error metric [25]. Through a simulation of a dataset in which the partially observed variable depended on the fully observed variables in a nonlinear way, MICE-RF showed less bias in parameter estimates and better confidence interval coverage. In our study, rf also performed well; however, the best performance was observed when pmm was used in the Cdiff cohort. Nonetheless, because the RMSEs were calculated across all laboratory variables, the improvement may be contributed by a few variables that were imputed better in perhaps some, but not all, cases. Further analysis will be needed to address this assumption.

The method presented here is an intuitive approach for any given complex disease where biosignatures or risk factors are only partially known and the relationship among the variables can be convoluted given the large dimensionality of the dataset. Even though the level of missingness can vary, the best results are typically obtained when the level of missingness is low or moderate. The improvement over conventional methods without the consideration of comorbidity information can be achieved when the missingness level is high. Our strategy was to ensure that (1) our experiment aligned with the current methodologies in practice and (2) others can easily adapt this modification to their work. In future directions, we will explore if advanced modeling frameworks such as the generative adversarial network [26] (GAN) or the newly proposed generative adversarial imputation nets (GAIN) framework [27] can be optimized for imputing laboratory values from EHRs.

Finally, our study provided a step in what we believe is a pipeline of data quality improvements for empowering machine learning models using EHRs. The main limitation of this approach is the need for large datasets. This is due to the nature of this approach, as the clustering step will reduce the sample size for the imputation, thus reducing its power. Therefore, this approach is ideal for machine learning applications where the sample size tends to be large and comprehensive. Our smallest cohort consisted of 10,000 OA patients. Our best prediction improvement was observed for the largest dataset of 46,215 patients. Another limitation of this study that we could not address is based on our masking strategy for the evaluation, which was done at random, even though we knew that the missingness in the EHR was not at random. However, given that we did not know *a priori* the reason for missingness for each patient, given the complex nature of the data, masking at random was the most sensible strategy in this case. As of now, we do not have a better strategy to simulate MNAR to withhold values. The contributing factors to MNAR are multifactorial and largely unknown.

This study had several other limitations. First, by converting the comorbidity information into binary, we may have lost important information. This study design can be enhanced further to answer a specific research question by optimizing the pattern of ICD codes recoded (both the frequency and time intervals) to capture the duration and severity of the conditions. Second, we withheld a relatively small number of values to evaluate our model. This is because we included laboratory codes with as high as 75% missingness and applied clustering prior to imputing; thus, withholding a higher level of laboratory values may further increase the sparsity of the dataset and introduce further bias. As a future direction, we plan on applying the algorithm several times to random subsamples of the data of size n/2 (n = number of samples). This repeated double randomization, similar to the concept of bagging and sub-bagging [28,29] algorithms, could further help optimize our strategy. Third, we are not limiting the window with respect to the diagnosis index event, as it should be for a carefully designed study [30,31]. However, the identification of pre- and post-index windows should be thoroughly planned based on the research question, the sparsity of the data, the healthcare system, and the variables under consideration [30]. However, as this is a proof-of-concept study, we did not limit our observation window in order to help improve our data availability so that we could experiment with different levels of missingness. Even though this is a limitation of this study, we showed what, in many instances, were only a few laboratory values for each patient for the less commonly used laboratory codes. Fourth, as this was a pilot study, we wanted to corroborate the generalizability and scalability of the proposed strategy. Therefore, we did not exhaustively vary the abstraction level nor the size of the clusters; however, we applied the model on three different cohorts that were created specifically for this study. Finally, by combining the laboratory codes into three groups (<25% missing, <50% missing, and <75% missing), we were unable to determine if this improvement was due to one or a few laboratory variables. Further assessments will be needed to study the improvement of imputation for each laboratory on a case-by-case basis for more targeted evaluations and improvements.

To conclude, the advantages of imputing missingness are manifold; imputation can be used for increasing the data density, improving the representation of data-poor patients, thus reducing the implicit algorithmic bias. Patients with limited access to healthcare and specialty care may be prone to be less-represented in models, because their data footprint is lower. The inclusion of more laboratory values is important as a prediction of a diagnosis; if it is not at least partially based on laboratory information, it could be weak. Predicting a future disease by only focusing on past diagnoses (i.e., using only information based on the ICD codes) is not taking full advantage of the information in electronic health records. Laboratory measurements, similar to imaging and imaging reports, are at the core of diagnosis and care management. The novelty of this study is in its intuitive design and relatively simple implementation in incorporating information from a patient’s comorbidity to improve the imputation of laboratory values.

As a future direction, we will investigate how best to impute longitudinal laboratory measures to better inform clinical studies. In addition, we will also explore integrating additional features, such as demographic information, age, gender, and medication usage, as well as genetic information when available, to further enhance the imputation outcome. Finally, we will evaluate various preprocessing and normalization strategies and evaluate if these manipulations can improve the outcome of our predictions, especially for variables with skewed distributions, and explore the impact of imputation on each laboratory value and further investigate any potential patterns or trends that can help improve predicting the missing values. To conclude, we optimized the level of abstraction needed to improve the imputation for three cohorts of varying sizes and complexities. This study demonstrates that the use of shared latent comorbidities can facilitate improvements in imputing laboratory measures from EHRs for downstream analysis and predictive modeling.

## Figures and Tables

**Table 1:** The root mean square error (RMSE) difference from imputation is applied with and without the integration of comorbidity information for the three datasets. Negative RMSE correspond to improvements by the hybrid approach. The predictive mean matching (pmm) and Random Forest (rf) model in Multivariate Imputation by Chained Equations (MICE) were used in this study. The reader is referred to Table A3, Table A4 and Table A5 for a more comprehensive results, with *p*-values reported from multiple runs.

*C. difficile* (Cdiff) Infection									
MICE-PMM					MICE-RF				
Cluster Number	Dimensionality Level (g)	Missingness < 25%	Missingness < 50%	Missingness < 75%	Cluster Number	Dimensionality Level (g)	Missingness < 25%	Missingness < 50%	Missingness < 75%
4	100	−0.77	7.12	−8.76	4	100	0.35	−1.47	−4.92
	1000	7.42	6.93	−31.47		1000	2.07	0.50	−12.72
	8160	−3.09	2.06	8.37		8160	−4.40	−3.28	0.49
8	100	0.11	9.19	12.39	8	100	1.40	11.06	−16.75
	1000	0.14	6.69	4.02		1000	1.24	4.04	9.73
	8160	4.63	10.09	6.99		8160	−0.88	−7.32	−5.11
16	100	−2.12	−3.00	5.03	16	100	−0.04	14.73	−2.36
	1000	5.92	16.21	23.33		1000	−0.19	5.98	−9.16
	8160	4.91	12.37	2.41		8160	0.63	−19.66	−9.50
**Inflammatory Bowel Disease (IBD)**									
**MICE-PMM**					**MICE-RF**				
**Cluster Number**	**Dimensionality Level (g)**	**Missingness < 25%**	**Missingness** **< 50%**	**Missingness < 75%**	**Cluster Number**	**Dimensionality Level (g)**	**Missingness < 25%**	**Missingness < 50%**	**Missingness < 75%**
2	100	0.94	0.22	−6.49	2	100	0.76	0.68	−3.19
	1000	1.28	0.08	5.44		1000	−1.14	0.23	−4.84
	7916	−0.89	1.97	0.24		7916	0.18	1.17	−8.35
4	100	1.26	0.17	−3.43	4	100	0.20	2.09	0.76
	1000	1.13	1.46	1.66		1000	−0.53	2.25	0.33
	7916	0.31	1.92	−4.15		7916	−0.91	1.97	−4.03
8	100	−0.36	2.85	6.60	8	100	0.97	−0.06	−4.16
	1000	−2.70	−0.74	−7.03		1000	1.08	2.15	1.17
	7916	0.01	4.40	3.76		7916	0.26	3.31	−8.24
**Osteoarthritis (OA)**									
**MICE-PMM**					**MICE-RF**				
**Cluster Number**	**Dimensionality Level (g)**	**Missingness < 25%**	**Missingness < 50%**	**Missingness < 75%**	**Cluster Number**	**Dimensionality Level (g)**	**Missingness < 25%**	**Missingness < 50%**	**Missingness < 75%**
4	100	0.04	0.08	−0.13	4	100	2.45	−4.23	6.83
	1000	0.03	0.11	−0.08		1000	3.35	10.16	−4.70
	2042	0.08	0.18	0.05		2042	1.70	−2.70	−0.75
8	100	−0.07	0.22	0.12	8	100	4.73	1.13	−0.10
	1000	−0.07	−0.07	0.16		1000	3.86	−1.27	−0.34
	2042	0.00	−0.01	−0.09		2042	4.42	−11.30	1.87
16	100	−0.02	0.10	0.20	16	100	−0.52	3.08	−2.33
	1000	0.08	0.15	−0.05		1000	1.41	−0.33	−6.45
	2042	−0.02	0.09	0.24		2042	1.60	3.23	10.93

## Data Availability

The data analyzed in this study is not publicly available due to privacy and security concerns. The data may be shared with a third party upon execution of data sharing agreement for reasonable requests, such requests should be addressed to V.A. (Vida Abedi) or R.Z.

## Appendix A

**Table A1:** Diagnosis codes used for inflammatory bowel disease and osteoarthritis.

Diagnosis	Inclusion Criteria Using ICD Codes
ICD9 Diagnosis: Crohn’s and Ulcerative Colitis	555, 55.0, 555.1, 555.2, 555.9, 556, 556.0, 556.1, 556.2, 556.3, 556.5, 556.6, 556.8, 556.9
ICD10 Diagnosis: Crohn’s and Ulcerative Colitis	K50.00, K50.011, K50.012, K50.013, K50.014, K50.018, K50.019, K50.10, K50.111, K50.112, K50.113, K50.114, K50.118, K50.119, K50.80, K50.811, K50.812, K50.813, K50.814, K50.818, K50.819, K50.90, K50.911, K50.912, K50.913, K50.914, K50.918, K50.919, K51.80, K51.00, K51.011, K51.012, K51.014, K51.018, K51.019, K51.20, K51.211, K51.212, K51.213, K51.218, K51.219, K51.30, K51.311, K51.313, K51.314, K51.318, K51.319, K51.411, K51.414, K51.419, K51.50, K51.511, K51.513, K51.514, K51.518, K51.519, K51.80, K51.811, K51.812, K51.813, K51.814, K51.818, K51.819, K51.90, K51.911, K51.912, K51.913, K51.914, K51.918, K51.919
ICD9 Diagnosis: Osteoarthritis	715; 715.0; 715.00; 715.09; 715.1; 715.10; 715.15; 715.16; 715.30; 715.35; 715.36; 715.8; 715.80; 715.85; 715.86; 715.89; 715.9; 715.90; 715.95; 715.96;
ICD10 Diagnosis: Osteoarthritis	M15.0; M15.9; M16.0; M16.10; M16.11; M16.12; M16.2; M16.30; M16.31; M16.32; M16.9; M17.0; M17.10; M17.11; M17.12; M17.9; M19.91

**Table A2:** Various summary statistics for the laboratory variables included in this study. The empty cell represents a percentage missing that is higher than 75%.

		Percentage Missing			Percent of Patient with 1 Lab Value			Average Number of Years between First and Last Laboratory Measurement, for Patient with 2 or More Measurements ( in Years)												Frequency of the Laboratory Measurements Calculated for Patients with Two or More Measurements											
LOINC ID	Short Description	Cdiff	IBD	OA	Cdiff	IBD	OA	Cdiff				IBD				OA				Cdiff				IBD				OA			
								Mean	Median	Q1	Q3	Mean	Median	Q1	Q3	Mean	Median	Q1	Q3	Mean	Median	Q1	Q3	Mean	Median	Q1	Q3	Mean	Median	Q1	Q3
**14957-5**	Microalbumin in Urine	75%	73%		31%	28%	26%	7.1	6.0	2.8	10.6	6.8	5.8	2.6	10.1	7.4	6.3	3.0	11.0	5	3	1	8	5	3	1	7	6	4	1	8
**14959-1**	Microalbumin/Creatinine in Urine		73%		31%	28%	25%	7.1	6.1	2.8	10.6	6.8	5.8	2.7	10.1	7.3	6.3	3.0	11.0	5	3	1	8	5	3	1	7	6	4	1	8
**13969-1**	Creatine kinase.MB in Serum/Plasma	54%	73%		26%	20%	24%	3.6	1.4	0.0	5.9	3.6	1.1	0.0	6.0	3.9	1.5	0.0	6.7	7	4	2	9	6	3	1	7	6	3	2	7
**18262-6**	Cholesterol in LDL in Serum/Plasma (by direct assay)	70%	67%		42%	38%	35%	5.5	4.7	2.1	8.2	5.5	4.6	1.9	7.9	5.9	5.2	2.3	8.7	4	2	1	5	3	2	1	4	4	2	1	5
**6768-6**	Alkaline phosphatase in Serum/Plasma	5%	11%		10%	6%	9%	8.5	7.8	3.1	13.4	8.9	8.2	3.5	13.7	10.1	9.7	4.9	15.1	18	11	5	22	14	8	3	18	14	9	4	17
**2284-8**	Folate in Serum/Plasma	65%	73%	74%	34%	33%	35%	3.2	1.1	0.0	5.1	3.5	1.4	0.0	5.6	3.4	1.2	0.0	5.5	3	2	1	3	3	2	1	3	3	2	1	3
**3024-7**	Thyroxine (T4) free in Serum/Plasma	60%	70%	74%	49%	45%	43%	5.7	4.2	1.4	8.9	6.0	4.4	1.7	9.4	6.3	4.6	1.8	9.8	3	2	1	4	3	2	1	3	4	2	1	4
**1798-8**	Amylase in Serum/Plasma	61%		73%	51%	50%	57%	3.9	2.3	0.2	6.5	4.5	3.1	0.8	7.2	4.5	3.0	0.4	7.7	3	1	1	3	3	1	1	3	2	1	1	2
**2502-3**	Iron saturation in Serum/Plasma	63%	73%	73%	45%	41%	41%	2.9	1.8	0.3	4.7	3.2	2.1	0.6	5.1	2.8	1.6	0.2	4.6	4	2	1	4	3	2	1	3	3	2	1	3
**27353-2**	Glucose mean value in Blood Estimated from glycated hemoglobin	59%	59%	72%	39%	33%	31%	4.6	4.2	1.8	7.2	4.5	4.1	1.8	7.3	4.9	4.7	2.1	7.8	7	3	1	9	6	2	1	7	7	3	1	10
**2157-6**	Creatine kinase in Serum/Plasma	46%	61%	71%	37%	25%	29%	4.4	2.6	0.1	7.3	4.5	2.8	0.2	7.5	4.9	3.4	0.2	8.0	7	4	1	8	5	2	1	5	5	3	1	6
**2340-8**	Glucose in Blood by Automated test strip	43%	62%	68%	23%	14%	22%	3.8	1.9	0.1	6.2	3.8	1.6	0.0	6.3	4.1	2.3	0.0	6.8	77	25	3	92	40	7	2	33	41	8	2	39
**17856-6**	Hemoglobin A1c/Hemoglobin.total in Blood	50%	50%	67%	37%	30%	29%	7.5	6.3	2.7	11.4	7.1	5.8	2.4	10.8	7.8	6.8	3.0	11.8	9	3	1	12	8	2	1	8	10	3	1	13
**2777-1**	Phosphate in Serum/Plasma	42%	60%	67%	28%	18%	29%	4.0	1.9	0.1	6.4	4.4	2.3	0.1	7.2	5.2	3.3	0.5	8.2	14	7	2	17	10	4	1	10	8	3	1	8
**19123-9**	Magnesium in Serum / Plasma	43%	66%	67%	30%	19%	33%	3.0	1.2	0.1	4.5	3.6	1.6	0.1	5.6	3.4	1.5	0.0	5.3	13	6	2	16	10	3	1	10	7	3	1	7
**2501-5**	Iron binding capacity.unsaturated in Serum/Plasma	52%	65%	64%	43%	38%	39%	4.2	2.8	0.6	6.5	4.8	3.2	1.1	7.3	4.3	2.8	0.6	6.5	4	2	1	4	3	2	1	4	3	2	1	4
**2276-4**	Ferritin in Serum/Plasma	55%	67%	63%	42%	42%	46%	4.5	3.2	0.9	6.8	4.9	3.6	1.3	7.3	4.7	3.4	1.1	7.1	4	2	1	4	4	2	1	4	3	2	1	3
**2132-9**	Cobalamin (Vitamin B12) in Serum/Plasma	51%	59%	61%	31%	29%	32%	4.5	2.8	0.2	7.2	5.1	3.1	0.4	8.3	4.9	3.3	0.2	7.8	3	2	1	4	4	2	1	4	3	2	1	4
**2498-4**	Iron in Serum/Plasma	50%	62%	60%	40%	36%	37%	4.4	3.0	0.7	6.7	5.0	3.5	1.2	7.5	4.5	3.1	0.6	6.9	4	2	1	4	4	2	1	4	3	2	1	4
**1988-5**	C reactive protein in Serum/Plasma	74%		58%	39%	56%	58%	3.6	2.3	0.5	5.8	4.5	3.5	1.3	7.0	3.8	2.3	0.5	5.8	3	1	1	3	4	2	1	5	2	1	1	2
**3040-3**	Lipase in Serum/Plasma	42%	69%	56%	38%	36%	45%	4.5	3.0	0.6	7.1	5.1	3.7	1.2	7.9	5.0	3.5	0.9	8.0	4	2	1	4	4	2	1	4	3	2	1	3
**13457-7**	Cholesterol in LDL in Serum/Plasma (by calculation)	28%	20%	40%	23%	19%	13%	9.5	8.9	4.2	14.5	9.4	8.8	4.0	14.7	10.7	10.7	5.5	16.0	8	5	2	11	7	4	2	9	10	7	3	14
**2085-9**	Cholesterol in HDL in Serum/Plasma	27%	19%	40%	22%	18%	13%	9.6	9.2	4.3	14.8	9.6	9.0	4.1	15.0	11.0	11.0	5.7	16.3	8	5	2	12	7	4	2	10	10	7	3	14
**1968-7**	Bilirubin.direct in Serum/Plasma	24%	43%	40%	25%	19%	26%	6.0	4.5	1.0	9.8	6.6	5.3	1.8	10.4	7.2	6.2	2.1	11.6	8	4	2	9	7	3	2	8	6	3	1	7
**2093-3**	Cholesterol in Serum/Plasma	26%	18%	39%	22%	18%	12%	10.0	9.6	4.5	15.5	10.0	9.6	4.2	15.7	11.4	11.5	5.9	16.9	9	5	2	12	8	4	2	10	10	7	3	15
**2571-8**	Triglyceride in Serum/Plasma	25%	19%	37%	21%	18%	13%	9.4	8.9	4.1	14.6	9.2	8.5	3.5	14.7	10.8	10.9	5.6	16.2	8	5	2	12	7	4	2	10	10	7	3	14
**1975-2**	Bilirubin.total in Serum/Plasma	5%	11%	11%	10%	6%	9%	8.4	7.6	3.0	13.1	8.8	8.1	3.5	13.5	9.9	9.5	4.8	14.8	17	11	5	22	14	8	3	17	14	9	4	17
**30239-8**	Aspartate aminotransferase in Serum/Plasma	5%	10%	10%	10%	6%	8%	8.8	8.1	3.2	13.9	9.1	8.5	3.7	14.1	10.4	10.2	5.2	15.7	19	12	5	24	15	9	3	19	15	10	4	20
**1743-4**	Alanine aminotransferase in Serum/Plasma	5%	10%	10%	10%	6%	8%	8.6	8.0	3.2	13.5	9.0	8.3	3.8	13.8	10.2	10.0	5.2	15.2	19	12	5	25	15	9	4	20	16	11	5	22
**2885-2**	Protein in Serum/Plasma	5%	10%	9%	10%	6%	9%	7.9	7.2	2.9	12.4	8.3	7.6	3.3	12.8	9.3	9.1	4.6	14.0	17	11	5	22	14	8	3	17	14	9	4	17
**10466-1**	Anion gap 3 in Serum/Plasma	6%	11%	8%	9%	3%	4%	6.3	6.3	2.5	10.2	6.4	6.4	2.7	10.4	7.3	7.9	3.9	11.1	39	25	10	51	22	11	4	27	26	16	7	32
**2028-9**	Carbon dioxide, total in Serum/Plasma	2%	4%	7%	7%	2%	3%	9.6	9.2	3.8	15.1	9.5	8.9	3.7	15.0	11.3	11.6	6.0	16.9	45	29	12	59	26	13	5	31	31	20	9	40
**2951-2**	Sodium in Serum/Plasma	2%	4%	7%	7%	2%	3%	9.6	9.2	3.8	15.2	9.6	9.1	3.8	15.1	11.3	11.7	6.0	16.9	45	30	12	60	26	13	5	32	31	20	9	40
**3094-0**	Urea nitrogen in Serum/Plasma	2%	4%	7%	7%	2%	3%	9.7	9.3	3.9	15.3	9.6	9.1	3.7	15.2	11.4	11.8	6.1	17.1	45	30	12	60	26	13	5	32	32	20	9	41
**17861-6**	Calcium in Serum/Plasma	2%	4%	6%	7%	2%	3%	8.8	8.5	3.5	14.0	8.9	8.5	3.5	14.0	10.4	10.8	5.5	15.6	44	29	12	58	25	13	5	31	30	19	9	38
**777-3**	Platelets in Blood	2%	6%	6%	6%	2%	4%	9.5	9.1	3.7	15.1	9.8	9.4	3.9	15.4	11.0	11.1	5.6	16.6	40	25	11	52	25	13	5	31	27	16	7	33
**789-8**	Erythrocytes in Blood	2%	6%	6%	6%	2%	4%	9.5	9.1	3.7	15.1	9.8	9.4	3.9	15.4	11.0	11.1	5.6	16.7	40	25	11	52	25	13	5	31	27	16	7	33
**788-0**	Erythrocyte distribution width	3%	6%	6%	6%	2%	4%	9.5	9.1	3.7	15.0	9.8	9.4	3.9	15.4	11.0	11.1	5.6	16.6	40	25	11	52	25	13	5	31	27	16	7	33
**6690-2**	Leukocytes in Blood	2%	6%	6%	6%	2%	4%	9.5	9.1	3.7	15.1	9.8	9.4	3.9	15.4	11.0	11.1	5.6	16.7	41	25	11	53	25	13	5	31	27	16	7	33
**2345-7**	Glucose in Serum/Plasma	2%	3%	6%	7%	2%	3%	9.7	9.4	3.9	15.4	9.7	9.2	3.8	15.4	11.5	12.0	6.1	17.3	46	30	13	61	27	14	5	32	32	21	9	41
**2075-0**	Chloride in Serum/Plasma	2%	4%	6%	7%	2%	3%	9.6	9.2	3.8	15.1	9.5	9.0	3.7	15.0	11.3	11.7	6.0	16.9	45	30	12	59	26	13	5	31	31	20	9	40
**32623-1**	Platelet mean volume in Blood	2%	6%	5%	7%	2%	4%	9.4	9.0	3.6	15.0	9.7	9.3	3.9	15.4	11.0	11.0	5.5	16.6	39	25	11	51	25	13	5	31	26	15	7	32
**2823-3**	Potassium in Serum/Plasma	2%	3%	5%	6%	2%	3%	9.7	9.3	3.9	15.4	9.6	9.1	3.7	15.2	11.5	11.8	6.1	17.2	47	31	13	62	27	14	5	33	32	21	9	41
**785-6**	MCH	2%	5%	5%	6%	2%	4%	9.5	9.1	3.7	15.0	9.8	9.4	3.9	15.4	11.0	11.1	5.6	16.6	40	25	11	52	25	13	5	31	27	16	7	33
**786-4**	MCHC	2%	5%	5%	6%	2%	4%	9.5	9.1	3.7	15.0	9.8	9.4	3.9	15.4	11.0	11.1	5.6	16.6	40	25	11	52	25	13	5	31	27	16	7	33
**2160-0**	Creatinine in Serum/Plasma	2%	3%	5%	6%	2%	3%	9.8	9.4	3.9	15.4	9.6	9.1	3.8	15.2	11.6	12.0	6.4	17.2	46	31	13	62	27	14	5	33	33	21	9	42
**718-7**	Hemoglobin in Blood	2%	4%	4%	6%	2%	3%	9.6	9.2	3.7	15.3	9.9	9.5	4.0	15.7	11.0	11.2	5.5	16.8	43	27	11	56	27	14	6	32	29	17	8	35
**4544-3**	Hematocrit of Blood by Automated count	2%	5%	4%	6%	2%	3%	9.5	9.2	3.7	15.2	9.8	9.4	3.9	15.5	11.0	11.1	5.5	16.7	42	26	11	55	26	14	5	32	28	16	8	34
**787-2**	Mean corpuscular volume, or MCV	2%	5%	4%	6%	2%	4%	9.5	9.1	3.7	15.1	9.8	9.4	3.9	15.4	11.0	11.1	5.6	16.7	40	25	11	52	25	13	5	31	27	16	7	33

**Table A3:** The RMSE difference from imputation is applied with and without the integration of comorbidity information for the Cdiff dataset. Negative RMSE correspond to improvement by the hybrid approach. The pmm and rf models in MICE were used in this study. The *p*-value is reported based on 10 runs.

			Cdiff-PMM		Cdiff-RF	
Missingness Level	Dimensionality Level (g)	Cluster Number	RMSE Difference	*p*-Value	RMSE Difference	*p*-Value
25%	100	4	−0.774	0.376	0.349	0.625
25%	100	8	0.110	0.739	1.402	0.532
25%	100	16	−2.121	0.306	−0.189	0.629
25%	1000	4	7.417	0.456	2.066	0.391
25%	1000	8	0.141	0.584	1.238	0.581
25%	1000	16	5.916	0.139	−0.035	0.233
25%	8160	4	−3.088	0.419	−4.397	0.582
25%	8160	8	4.628	0.150	−0.882	0.868
25%	8160	16	4.910	0.493	0.631	0.594
50%	100	4	7.117	0.459	−1.470	0.789
50%	100	8	9.189	0.759	11.064	0.796
50%	100	16	−3.005	0.351	14.731	0.472
50%	1000	4	6.934	0.920	0.503	0.675
50%	1000	8	6.695	0.230	4.044	0.432
50%	1000	16	16.207	0.087	5.976	0.196
50%	8160	4	2.060	0.481	−3.279	0.865
50%	8160	8	10.087	0.435	−7.323	0.502
50%	8160	16	12.366	0.190	−19.655	0.476
75%	100	4	−8.756	0.386	−4.916	0.662
75%	100	8	12.386	0.174	−16.748	0.487
75%	100	16	5.026	0.392	−2.362	0.513
75%	1000	4	−31.468	0.017	−12.722	0.982
75%	1000	8	4.024	0.266	9.729	0.405
75%	1000	16	23.333	0.139	−9.162	0.258
75%	8160	4	8.368	0.569	0.488	0.787
75%	8160	8	6.993	0.515	−5.113	0.631
75%	8160	16	2.414	0.957	−9.496	0.979

**Table A4:** The RMSE difference from imputation is applied with and without the integration of comorbidity information for the IBD dataset. Negative RMSE correspond to improvement by the hybrid approach. The pmm and rf models in MICE were used in this study. The *p*-value is reported based on 10 runs.

			IBD-PMM		IBD-RF	
Missingness Level	Dimensionality Level (g)	Cluster Number	RMSE Difference	*p-*Value	RMSE Difference	*p*-Value
25%	100	2	0.938	0.565	0.756	0.759
25%	100	4	1.264	0.948	0.200	0.695
25%	100	8	−0.359	0.273	0.969	0.339
25%	1000	2	1.284	0.583	−1.145	0.425
25%	1000	4	1.134	0.234	−0.526	0.733
25%	1000	8	−2.696	0.196	1.083	0.132
25%	7916	2	−0.886	0.974	0.176	0.944
25%	7916	4	0.313	0.210	−0.906	0.249
25%	7916	8	0.005	0.307	0.264	0.177
50%	100	2	0.218	0.336	0.682	0.448
50%	100	4	0.168	0.196	2.094	0.281
50%	100	8	2.851	0.072	−0.057	0.428
50%	1000	2	0.080	0.411	0.230	0.561
50%	1000	4	1.465	0.601	2.246	0.569
50%	1000	8	−0.745	0.609	2.145	0.604
50%	7916	2	1.973	0.338	1.165	0.912
50%	7916	4	1.922	0.188	1.973	0.676
50%	7916	8	4.401	0.078	3.309	0.288
75%	100	2	−6.485	0.256	−3.192	0.447
75%	100	4	−3.428	0.632	0.756	0.580
75%	100	8	6.598	0.825	−4.165	0.624
75%	1000	2	5.436	0.721	−4.835	0.306
75%	1000	4	1.664	0.511	0.329	0.584
75%	1000	8	−7.031	0.581	1.175	0.771
75%	7916	2	0.239	0.378	−8.353	0.175
75%	7916	4	−4.155	0.470	−4.033	0.689
75%	7916	8	3.760	0.468	−8.244	0.096

**Table A5:** The RMSE difference from imputation is applied with and without the integration of comorbidity information for the OA dataset. Negative RMSE correspond to improvement by the hybrid approach. The pmm and rf models in MICE were used in this study. The *p*-value is reported based on 10 runs.

			OA-PMM		OA-RF	
Missingness Level	Dimensionality Level (g)	Cluster Number	RMSE Difference	*p-*Value	RMSE Difference	*p*-Value
25%	100	4	0.035	0.317	2.449	0.245
25%	100	8	−0.074	0.444	4.734	0.385
25%	100	16	−0.017	0.375	−0.518	0.525
25%	1000	4	0.035	0.687	3.351	0.247
25%	1000	8	−0.066	0.363	3.859	0.183
25%	1000	16	0.085	0.706	1.414	0.172
25%	2042	4	0.081	0.889	1.705	0.161
25%	2042	8	0.004	0.595	4.417	0.460
25%	2042	16	−0.019	0.202	1.602	0.810
50%	100	4	0.081	0.700	−4.229	0.199
50%	100	8	0.218	0.079	1.132	0.970
50%	100	16	0.101	0.087	3.082	0.357
50%	1000	4	0.106	0.653	10.161	0.843
50%	1000	8	−0.066	0.577	−1.271	0.480
50%	1000	16	0.147	0.620	−0.328	0.891
50%	2042	4	0.178	0.252	−2.703	0.946
50%	2042	8	−0.013	0.216	−11.300	0.409
50%	2042	16	0.092	0.643	3.229	0.376
75%	100	4	−0.131	0.186	6.828	0.213
75%	100	8	0.118	0.507	−0.098	0.434
75%	100	16	0.197	0.142	−2.326	0.889
75%	1000	4	−0.077	0.092	−4.702	0.222
75%	1000	8	0.157	0.428	−0.343	0.653
75%	1000	16	−0.053	0.508	−6.447	0.651
75%	2042	4	0.055	0.649	−0.749	0.430
75%	2042	8	−0.089	0.549	1.865	0.768
75%	2042	16	0.237	0.014	10.926	0.061
